# Impact of parturition induction, farrowing environment and birth weight class on endocrine and metabolic plasma parameters related to piglet vitality

**DOI:** 10.1186/s12917-025-04845-2

**Published:** 2025-06-07

**Authors:** Heinke Lickfett, Michael Oster, Andreas Vernunft, Henry Reyer, Eduard Muráni, Solvig Görs, Cornelia C. Metges, Hartwig Bostedt, Klaus Wimmers

**Affiliations:** 1 Research Institute for Farm Animal Biology (FBN), 18196 Dummerstorf, Germany; 2 Veterinary Clinic for Reproductive Medicine and Neonatology, Justus-Liebig-University Gießen, 35392 Gießen, Germany; 3 Chair of Animal Breeding and Genetics, Faculty of Agricultural and Environmental Sciences, University Rostock, 18059 Rostock, Germany

**Keywords:** Newborn, Piglet survival, Farrowing management, Housing, Neonatal adaptation, Prematurity

## Abstract

**Background:**

High pre-weaning mortality rate in pig husbandry is not acceptable across all production systems. Successful neonatal adaptation is key to improve piglet vitality and survival, which could be increased through adequate management practices around parturition. In this study, vitality parameters in German Landrace piglets (*n* = 96) are evaluated based on (i) the use or omission of hormonal parturition induction and (ii) the farrowing environment, i.e., crates (1.0 m^2^) or pens (6.5 m^2^). In addition, the effects of (iii) the allocation to birth weight classes were analysed. The aim was to quantify plasma metabolites with significance for organ maturation and energy utilization in neonates and suckling piglets with different birth weights as a function of the hormonal induction of parturition and farrowing environment in a longitudinal approach.

**Results:**

Farrowing traits including litter size, stillborn piglets, and inter-farrowing intervals did not differ due to parturition induction nor farrowing environment (*P* > 0.05). Piglets from spontaneous parturitions required less time until the first suckling event (*P* < 0.05) and had higher rectal temperatures after 24 h than piglets from induced parturition (*P* < 0.05). Spontaneously born piglets exhibited lower plasma glucose (1d; 4d) and lactate levels (4d), but higher levels of NEFA (4d), total protein (4d; 20d), and blood urea nitrogen (1d; 20d) compared to piglets from induced parturitions (*P* < 0.05). This suggests higher organ maturation and that proteins are probably utilised primarily for growth rather than energy production during the neonatal adaptation phase, i.e., the first four days *postnatum*. Pen-born piglets exhibited lower glucose (1d; 4d), lower inositol (0.5–6.0 h; 1d), higher urea (0.5–6.0 h), and higher creatinine levels (0.5–6.0 h; 1d; 29d) compared to piglets from crated dams (*P* < 0.05), suggesting differences in energy metabolism, renal function, and physical activity between the two farrowing environments. Hypotrophic piglets showed lower plasma glucose concentrations (1d; 4d) and higher cortisol (1d; 4d) and NEFA levels (4d) indicating lipid mobilisation and induction of gluconeogenesis via muscle protein catabolism (*P* < 0.05).

**Conclusion:**

Sow management, i.e., the induced parturition using exogenous hormones as well as the applied farrowing environment are reflected in plasma metabolite profiles of piglets and must be critically questioned in routine use with regard to the effects on piglet development.

**Supplementary Information:**

The online version contains supplementary material available at 10.1186/s12917-025-04845-2.

## Background

The successful neonatal adaptation of a piglet is key to survival and metabolic health and takes place in three distinct phases as reviewed elsewhere [1]. Immediately after birth, the piglet undergoes critical physiological transitions involving respiratory and cardiovascular systems. In fact, the first breath not only initiates lung function, but also triggers a cascade of cardiovascular adaptations that are necessary for early survival [1]. During the first 24 h, the piglet must be able to independently regulate its body temperature and acid-base balance [1]. Movements need to be coordinated to enable colostrum intake and to start an independent digestion that ensures nutrient absorption [1]. Subsequently, organs such as the kidneys and liver mature until the 14th day *postnatum* [1]. The neurological, immunological, and muscular functions are thought to be developed until 28 days *postnatum* [1]. However, neonatal adaptation proves to be insufficient for some piglets. In fact, the pre-weaning mortality is ranging from 12.4 to 21.9% found in studies with conventional conditions [2–4], thereby leading to serious financial and ethical problems. Most losses occur in the early postnatal adaptation phase, i.e., within the first three to four days of life [5–7].

The farrowing environment used in the peripartal period, i.e. conventional farrowing crates and farrowing pens, has been discussed as a factor influencing pre-weaning mortality. In some studies, a higher mortality was observed in pen housing (13.7 − 26.8%) compared to crate housing (11.8 − 17.0%) [8–10], while others did not reveal any difference in pre-weaning mortality rate between the different farrowing environments [11]. Clearly, these inconsistent results indicate the need for further investigations, especially as the causes of death are often not clearly stated. The farrowing process influences the neonatal outcome of the piglets [12], as shown by the stillbirth rate as a function of the farrowing duration. Hormonal parturition induction can shorten the total farrowing duration compared to spontaneous, non-induced farrowings in pigs [13]. However, in other studies, no effects of parturition induction on total farrowing duration was observed [14, 15], but a higher frequency of manual interventions was documented [15, 16]. In fact, the effect of standard parturition induction though hormone administration on frequency of manual intervention and animal welfare issues were rarely studied [17]. Additionally, parturition induction significantly reduces the physiological gestation length about 1–3 days depending on sow genetics [18]. Therefore, it is conceivable that a number of piglets remain immature at the time of hormonal parturition induction, which may ultimately reduce the success of neonatal adaptation and further development [1]. This applies in particular to the maturation of the kidney and liver, which might not yet be completed at the predetermined time of parturition induction. Piglet vitality is influenced by many factors such as genetics, placental vascularization, farrowing process, and birth weight [19, 20]. As litter sizes have increased during the last decades, the number of piglets born with a low birth weight has continued to rise [5, 21, 22]. Piglets with low birth weight have increased latency to first suckling [23] and have the disadvantage of competing for teats with heavier littermates [24]. Additionally, low birth weight piglets have higher heat loss due to their unfavourable surface-to-volume ratio [25] and therefore require larger amounts of energy relative to their body weight compared to littermates with a higher birth weight [26]. As a result, a relatively larger proportion of low birth weight piglets die before weaning compared with heavier littermates [5, 23, 27, 28].

We hypothesise that housing sows in pens or crates during parturition and the first four days of lactation influences the vitality and physical activity of piglets. Furthermore, we expect differences in piglet vitality based on whether they were born via induced or spontaneous parturition, potentially leading to variations in the maturity of their organ systems at birth. To this end, we conducted a longitudinal study to quantify clinical-chemical blood parameters and metabolites that reflect the vitality of neonatal and suckling piglets in relation to (i) parturition induction and labour stimulation by hormone administration, (ii) sow farrowing environment, and (iii) birth weight category. This research is part of a larger study that has already published results on an additional experimental factor, i.e. pig breed [29].

## Results

### Farrowing and litter characteristics

The average parity of the 18 sows monitored in this study was 4.0 ± 0.32 (mean ± SEM). The gestation length for spontaneous parturitions was 115.25 ± 0.63 days *post-conceptionem* (dpc) in crates and 115.25 ± 0.75 dpc in pens. All sows with induced parturition farrowed on 115 dpc. As stated in Table 1, farrowing traits did not differ due to parturition induction nor the farrowing environment in this experimental setting. Analyses revealed a significant interaction of parturition induction and farrowing environment on time until first suckling. Moreover, piglets from spontaneous parturition required less time until first suckling (30.02 ± 2.25 min) than piglets from induced parturitions (35.94 ± 2.32 min; *P* = 0.002). Hypotrophic piglets were more likely to die in the first three days (*P* < 0.001; 16 losses of 70 born alive piglets) and also during the entire suckling period (*P* < 0.001; 24 losses of 70 born alive piglets) than non-hypotrophic control piglets (day 1: 13 losses of 213 born alive piglets; day 14: 20 losses of 213 born alive piglets).

**Table 1:** Farrowing and litter characteristics of German Landrace (GL). The data were collected from spontaneous and induced parturitions in farrowing crates and farrowing pens. Data make use of entire litters and are presented as mean ± sem. Data on induced parturitions also have been analysed in a companion study [29]. Significant effects are highlighted in bold (*P* < 0.05)

Item	Spontaneous parturition		Induced parturition		*P*-value parturition induction	*P*-value farrowing environment	*P*-Value parturition induction × farrowing environment
	Farrowing crate	Farrowing pen	Farrowing crate	Farrowing pen			
No. of observed litters	4	4	5	5			
Farrowing duration [min]	345.25 ± 43.73	283.25 ± 62.35	267.60 ± 39.95	284.50 ± 51.50	0.265	0.841	0.480
Inter-farrowing interval [min]	21.16 ± 2.07	18.06 ± 4.14	17.80 ± 3.34	15.68 ± 2.10	0.456	0.664	0.884
Litter size [n]	17.25 ± 1.31	17.00 ± 1.87	16.40 ± 1.29	19.00 ± 0.32	0.639	0.142	0.275
Litter weight [kg]	21.42 ± 1.88	21.18 ± 1.00	20.64 ± 0.49	21.34 ± 2.60	0.760	0.772	0.794
Live-born piglets [n]	16.00 ± 1.22	15.00 ± 0.71	16.00 ± 1.34	15.80 ± 1.50	1.000	0.910	0.763
Stillborn piglets [n]	1.25 ± 0.48	2.00 ± 1.35	0.40 ± 0.24	3.20 ± 1.53	0.587	0.073	0.359
Live-born piglets [%]	92.82 ± 2.84	90.15 ± 5.98	97.50 ± 1.53	83.26 ± 8.07	0.659	0.199	0.516
Stillborn piglets [%]	7.18 ± 2.84	9.85 ± 5.98	2.50 ± 1.53	16.74 ± 8.07	0.659	0.199	0.516
Mummies [n]	0	0.25 ± 0.25	0.40 ± 0.24	0.20 ± 0.20	0.200	0.487	0.303
Hypotrophic piglets [n]	4.00 ± 0.91	3.00 ± 0.41	4.80 ± 2.42	4.00 ± 1.00	0.720	0.704	0.949
Eutrophic piglets [n]	6.50 ± 1.32	6.50 ± 1.71	7.20 ± 1.50	5.80 ± 0.80	0.720	0.450	0.613
Hypertrophic piglets [n]	5.25 ± 1.70	5.50 ± 1.50	4.20 ± 2.08	6.60 ± 1.54	0.682	0.327	0.554
Vitality score^1^	6.81 ± 0.16	6.51 ± 0.13	6.96 ± 0.12	6.67 ± 0.13	0.454	0.144	0.942
Time until first suckling [min]	24.45 ± 2.65	36.05 ± 3.57	39.74 ± 3.72	33.02 ± 2.95	**0.002**	0.168	**0.007**
Pre-weaning mortality [%]	12.75 ± 2.56	6.25 ± 2.46	14.60 ± 5.50	22.00 ± 3.67	0.513	0.600	0.252

^1^ The assessment included breathing, skin colour, standing attempts, and meconium staining

### Rectal temperature

Piglets from spontaneous births had higher rectal temperatures (38.3 ± 0.1 °C) compared to piglets of induced parturitions (37.8 ± 0.1 °C) on day 1 (*P* = 0.025), but not at 0.5–6.0 h *postnatum* (*P* > 0.05). Hypotrophic piglets had lower rectal temperatures (37.2 ± 0.2 °C) than non-hypotrophic piglets (37.5 ± 0.1 °C) at 0.5–6.0 h after birth (*P* = 0.022), but not at day 1 (*P* > 0.05). The farrowing environment did not affect the rectal temperature (*P* > 0.05).

### Body weight evaluation

Depending on the assignment to the birth weight classes after parturition, the body weight of the piglets remained different between the distinct birth weight classes throughout early postnatal development until weaning. This was supported by repeated measurement approach (*P* < 0.001) as well as by analyses per time-point (Table 2). When analysing the weight data using repeated observations, an effect of parturition induction (*P* = 0.562), farrowing environment (*P* = 0.976), and their two-way interaction (*P* = 0.802) was not present. However, when looking at individual time points, the effect of parturition induction became evident at day 20 (*P* = 0.004) and day 29 (*P* = 0.026) with lower body weights of piglets from spontaneous parturitions compared to induced parturitions (Table 2). Piglets born in the pen had lower body weights than piglets from a crated dam at day 20 (*P* = 0.018).

**Table 2:** Body weight of neonates and suckling piglets at 0.5–6.0 h, day 1, day 4, day 20 and day 29. The data were collected from selected German Landrace piglets (*n* = 96) affiliated with distinct birth weight classes from induced and spontaneous parturition in farrowing crates and farrowing pens. Data on induced parturitions also have been analysed in a companion study [29]. Data are presented as mean ± sem. Significant effects are highlighted in bold (*P* < 0.05)

Time point	Parturition induction		Farrowing environment		Birth weight class	
	Induced (*n* = 46)	Spontaneous (*n* = 46)	Crate (*n* = 46)	Pen (*n* = 46)	800–1100 g (*n* = 32)	> 1100 g (*n* = 64)
0.5–6.0 h [kg]	1.36 ± 0.04	1.38 ± 0.05	1.35 ± 0.05	1.38 ± 0.05	**1.02 ±0.02** ^**§**^	**1.53 ±0.03** ^**$**^
1 d [kg]	1.36 ± 0.05	1.45 ± 0.05	1.41 ± 0.05	1.40 ± 0.05	**1.04 ±0.02** ^**§**^	**1.58 ±0.03** ^**$**^
4 d [kg]	1.88 ± 0.07	1.81 ± 0.07	1.86 ± 0.07	1.82 ± 0.06	**1.36 ±0.04** ^**§**^	**2.07 ±0.04** ^**$**^
20 d [kg]	**6.08 ±0.19** ^**a**^	**5.41 ±0.17** ^**b**^	**5.85 ±0.21** ^**A**^	**5.63 ±0.17** ^**B**^	**4.66 ±0.19** ^**§**^	**6.21 ±0.14** ^**$**^
29 d* [kg]	**7.79 ± 0.26** ^**a**^	**6.98 ±0.22** ^**b**^	7.42 ± 0.27	7.31 ± 0.22	**6.15 ±0.30** ^**§**^	**7.91 ±0.17** ^**$**^

* *post*-weaning; different superscripts indicate statistical significance per individual time point (*P* < 0.05) due to parturition induction (^a, b^), farrowing environment (^A, B^), and birth weight class (^§,$^)

### Repeated measurements of plasma metabolites and hormones

The three-way interaction for parturition induction, farrowing environment, and birth weight class was significant for plasma chloride levels (*P* = 0.010). Respective mean ± SEM per experimental group are displayed in Additional Table S1. There was a significant two-way interaction between the parturition induction and farrowing environment for urea (*P* = 0.032) and creatinine (*P* < 0.001). The two-way interaction of parturition induction and birth weight class was significant for lactate (*P* = 0.046) and glucose (*P* = 0.038). The two-way interaction between the farrowing environment and birth weight class was not significant for any of the analysed plasma metabolites.

Main effects were evaluated via repeated measurements analysis using data from 96 selected piglets (Table 3). The parturition induction impacted the plasma levels of lactate (*P* < 0.001), glucose (*P* = 0.035) and total protein (*P* < 0.001), as well as creatinine (*P* = 0.009), ammonia (*P* < 0.001), triiodothyronine (*P* = 0.040), cortisol (*P* = 0.031), sodium (*P* < 0.001), and uric acid (*P* < 0.001). The farrowing environment showed effects on the plasma levels of insulin (*P* = 0.023), glucose (*P* < 0.001), creatinine (*P* < 0.001), and cortisol (*P* = 0.004). Birth weight class had an effect on the plasma concentrations of insulin (*P* = 0.047), glucose (*P* = 0.002), inositol (*P* < 0.001), NEFA (*P* = 0.027), blood urea nitrogen (*P* = 0.001), uric acid (*P* = 0.002), cortisol (*P* = 0.032), and sodium (*P* = 0.025). Moreover, a time effect was observed for plasma levels of NEFA (*P* = 0.016), triglycerides (*P* = 0.005), total protein (*P* = 0.004), albumin (*P* < 0.001), blood urea nitrogen (*P* = 0.001), uric acid (*P* < 0.001), cortisol (*P* = 0.014), and sodium (*P* = 0.001).

**Table 3:** Level of significance for the analysed data of plasma metabolites and hormones due to parturition induction, farrowing environment and birth weight class following the repeated measurements approach. The data were obtained from German Landrace piglets (*n* = 96) of different birth weight classes from spontaneous and induced parturitions which born in crates and pens. The analyses include data from 1–5 time points per parameter. Significant effects are highlighted in bold (*P* < 0.05)

Item	Parturition induction	Farrowing environment	Birth weight class	Time	I × F	Number of time points^1^
Albumin	0.265	0.289	0.452	**< 0.001**	0.855	5
Ammonia	**< 0.001**	0.083	0.631	0.324	0.224	5
Chloride	0.173	0.576	0.579	0.091	0.842	5
Cortisol	**0.031**	**0.004**	**0.032**	**0.014**	0.882	5
Creatinine	**0.009**	**< 0.001**	0.596	0.051	**< 0.001**	5
Fructose	0.361	0.421	0.077	-	0.182	1
Glucose	**0.035**	**< 0.001**	**0.002**	0.248	0.423	3
Haptoglobin	0.480	0.405	0.479	0.112	0.442	5
Inositol	0.889	0.113	**< 0.001**	0.741	0.753	2
Insulin	0.476	**0.023**	**0.047**	0.161	0.066	3
Lactate	**< 0.001**	0.488	0.616	0.951	0.529	3
NEFA	0.089	0.495	**0.027**	**0.016**	0.661	5
Sodium	**< 0.001**	0.160	**0.025**	**0.001**	0.205	5
Total protein	**< 0.001**	0.729	0.051	**0.004**	0.570	5
Triglycerides	0.265	0.119	0.628	**0.005**	0.199	5
Triiodothyronine	**0.040**	0.774	0.453	0.494	0.074	2
Blood urea nitrogen	0.470	0.266	**0.001**	**0.001**	**0.032**	5
Uric acid	**< 0.001**	0.950	**0.002**	**< 0.001**	0.907	5

^1^ Additional Table S2 lists the time points when each parameter was analysed; NEFA – non-esterified fatty acids; I – Parturition induction; F – Farrowing environment

### Evaluation of plasma metabolites and hormones per time point

The three-way interaction including parturition induction, farrowing environment, and birth weight class was significant for total protein on day 20 (*P* = 0.014) and for haptoglobin on day 1 (*P* = 0.013). It was also significant for sodium on day 20 (*P* = 0.015) and day 29 (*P* = 0.043) and for chloride on day 4 (*P* = 0.013; Additional Table S1).

A significant two-way interaction between parturition induction and farrowing environment was observed for glucose on day 1 (*P* = 0.040) and for total protein on day 20 (*P* = 0.049). This interaction was also significant for plasma creatinine levels during the neonatal adaptation period (0.5–6.0 h: *P* = 0.003; day 1: *P* < 0.001; day 4: *P* < 0.001). Significant two-way interactions between parturition induction and farrowing environment were also noted for albumin on day 1 (*P* = 0.024) and for blood urea nitrogen at 0.5–6.0 h (*P* = 0.002) and on day 20 (*P* = 0.041). Additionally, this interaction was significant for sodium levels on day 20 (*P* = 0.028; Additional Table S3; Additional Table S4).

Significant two-way interactions were found between parturition induction and birth weight class for glucose (*P* = 0.020), haptoglobin (*P* = 0.0021), and cortisol (*P* = 0.027) on day 1. The interaction was also significant for sodium at the time points 0.5–6.0 h (*P* = 0.012), day 20 (*P* = 0.049), and day 29 (*P* = 0.048).

Significant two-way interactions were found between farrowing environment and birth weight class for inositol on day 1 (*P* = 0.021) as well as for total protein at 0.5–6.0 h (*P* = 0.045). For haptoglobin, a significant two-way interaction was found on day 1 (*P* = 0.024) and day 20 (*P* = 0.048). The interaction between the farrowing environment and birth weight class was significant for sodium on day 1 (*P* = 0.013), day 20 (*P* = 0.031) and day 29 (*P* = 0.004), and for chloride on day 4 (*P* = 0.015).

**Fig. 1:**
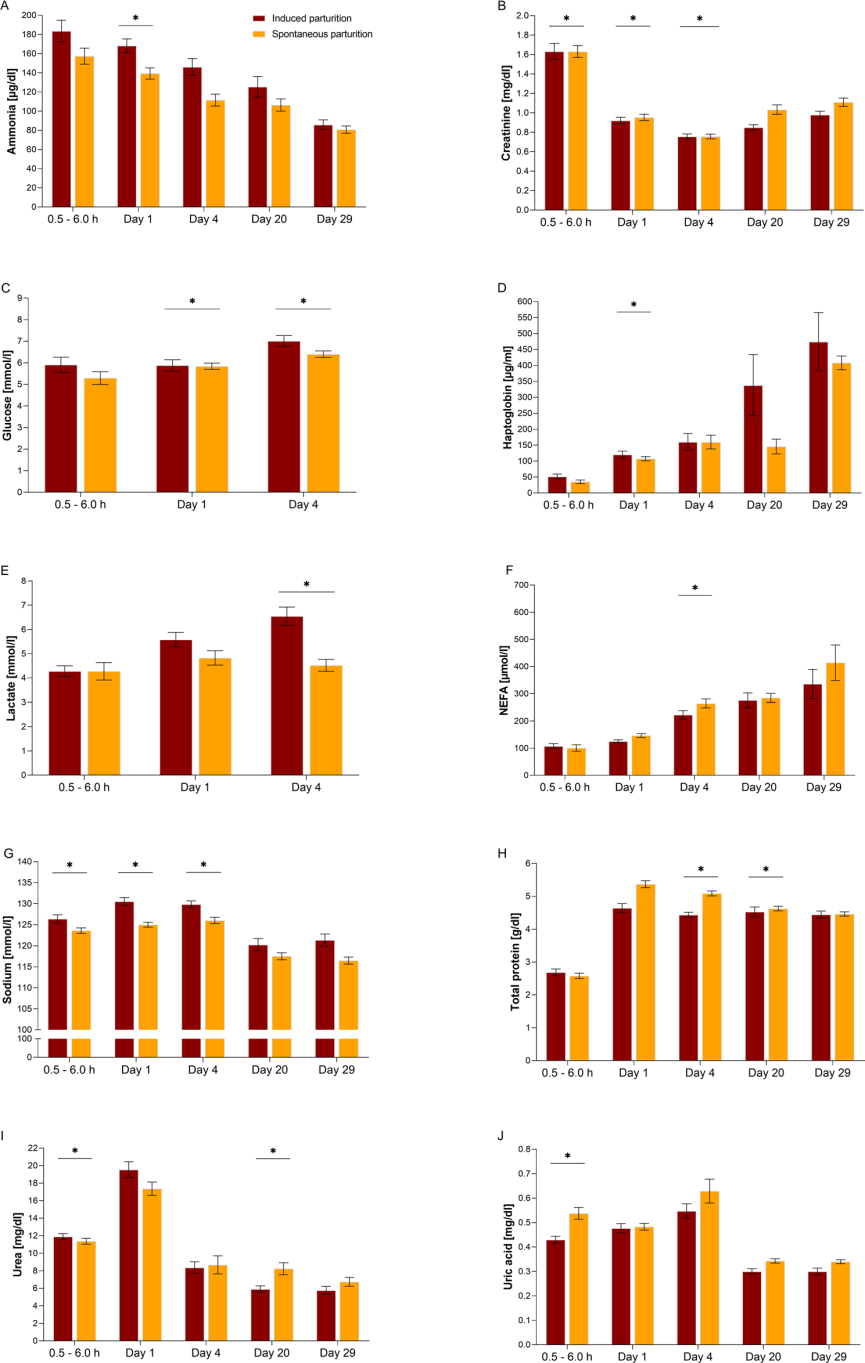
Effect of induced vs. spontaneous parturition on plasma metabolites in neonatal and suckling piglets. Plasma metabolites exhibiting significant differences for specific time points comprised (**A**) ammonia, (**B**), creatinine, (**C**), glucose, (**D**) haptoglobin, (**E**) lactate, (**F**) NEFA (non-esterified fatty acids), (**G**) sodium, (**H**) total protein, (**I**) blood urea nitrogen, and (**J**) uric acid. Samples were taken at 0.5–6.0 h, day 1, day 4, day 20, and day 29. Data on induced parturitions also have been analysed in a companion study [29]. Significant differences (*P* < 0.05) are indicated by asterisks

As shown in Fig. 1, piglets from spontaneous parturition had 31% lower plasma lactate levels than piglets from hormonally induced parturition on day 4 (*P* = 0.016). Compared to piglets from induced parturition, the glucose concentrations of the piglets from spontaneous parturition were significantly lower on day 1 (*P* = 0.026) and day 4 (*P* = 0.10). Piglets from spontaneous parturition had 19% higher NEFA concentrations compared to piglets from induced parturition on day 4 (*P* = 0.013). Total protein concentrations were also elevated in piglets from spontaneous parturition on day 4 (*P* = 0.004) and day 20 (*P* = 0.024). Blood urea nitrogen concentrations were lower in piglets from spontaneous parturition than in piglets from induced parturition at 0.5–6.0 h of age (*P* = 0.041), and were 39% higher on day 20 (*P* = 0.002). Piglets from spontaneous parturition had lower creatinine levels at 0.5–6.0 h (*P* = 0.004) and higher creatinine levels on day 1 (*P* < 0.001) and day 4 (*P* < 0.001) than piglets from induced parturition. Ammonia levels were 17% lower in spontaneously born piglets than in piglets from induced parturition on day 1 (*P* = 0.003). The plasma uric acid concentrations were 25% higher in piglets born spontaneously compared to piglets from induced parturition at 0.5–6.0 h (*P* = 0.007). Haptoglobin levels were 11% lower in piglets from spontaneous parturition on day 1 (*P* = 0.041). Spontaneously born piglets had lower plasma sodium concentrations during the neonatal adaptation period than piglets from induced parturition (0.5–6.0 h: *P* = 0.023; day 1: *P* = 0.001; day 4: *P* = 0.019; Additional Table S3; Additional Table S4).

**Fig. 2:**
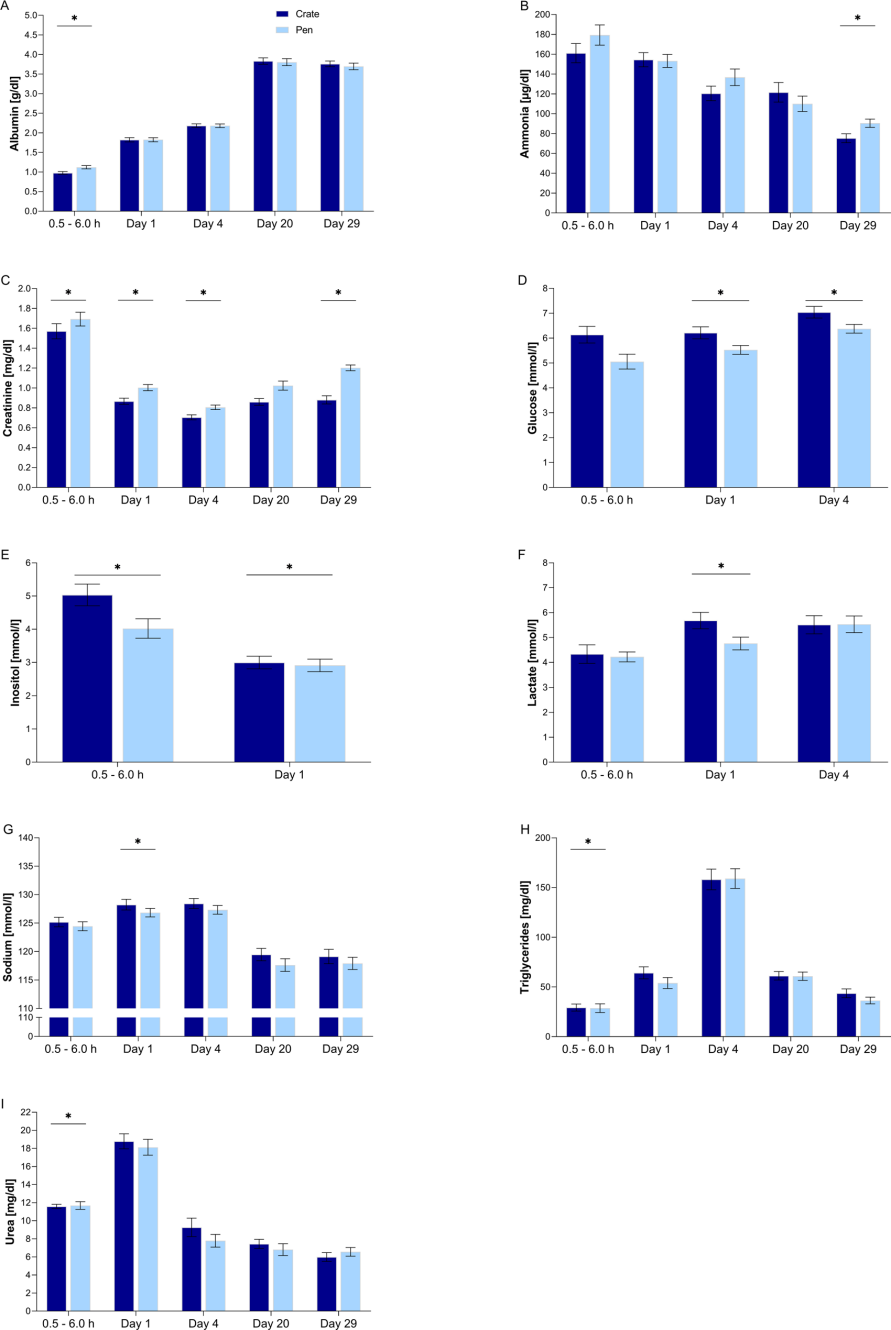
Effect of farrowing environment, i.e. pens or crates on plasma metabolites in neonatal and suckling piglets. Plasma metabolites exhibiting significant differences for specific time points comprised (**A**) albumin, (**B**) ammonia, (**C**) creatinine, (**D**) glucose, (**E**) inositol, (**F**) lactate, (**G**) sodium, (**H**) triglycerides, and (**I**) blood urea nitrogen. Samples were taken at 0.5–6.0 h, day 1, day 4, day 20, and day 29. Data on parturitions from crates also have been analysed in a companion study [29]. Significant effects (*P* < 0.05) are indicated by asterisks

As shown in Fig. 2, piglets born in the pen had 16% lower plasma lactate levels than piglets from a crated dam on day 1 (*P* = 0.027). Lower plasma glucose levels were found in piglets in the pen compared to piglets with a crated dam on day 1 (*P* = 0.006) and day 4 (*P* = 0.009). Inositol levels were reduced in piglets in the pen compared to piglets with crated dam at 0.5–6.0 h (*P* = 0.023) and on day 1 (*P* = 0.038). Piglets in the pen had lower triglyceride (*P* = 0.033) concentrations and higher urea concentrations (*P* = 0.016) than piglets with crated dams at time 0.5–6.0 h. In the pen, piglets had higher plasma creatinine concentrations than piglets with crated dams except for day 20 (0.5–6.0 h: *P* = 0.003; day 1: *P* < 0.001; day 4: *P* < 0.001; day 29: *P* < 0.001). Ammonia levels were 20% higher in piglets raised in the pen at day 29 (*P* = 0.044). Piglets in the pen had lower plasma sodium levels than piglets with a crated dam on day 1 (*P* = 0.035; Additional Table S3; Additional Table S4).

**Fig. 3:**
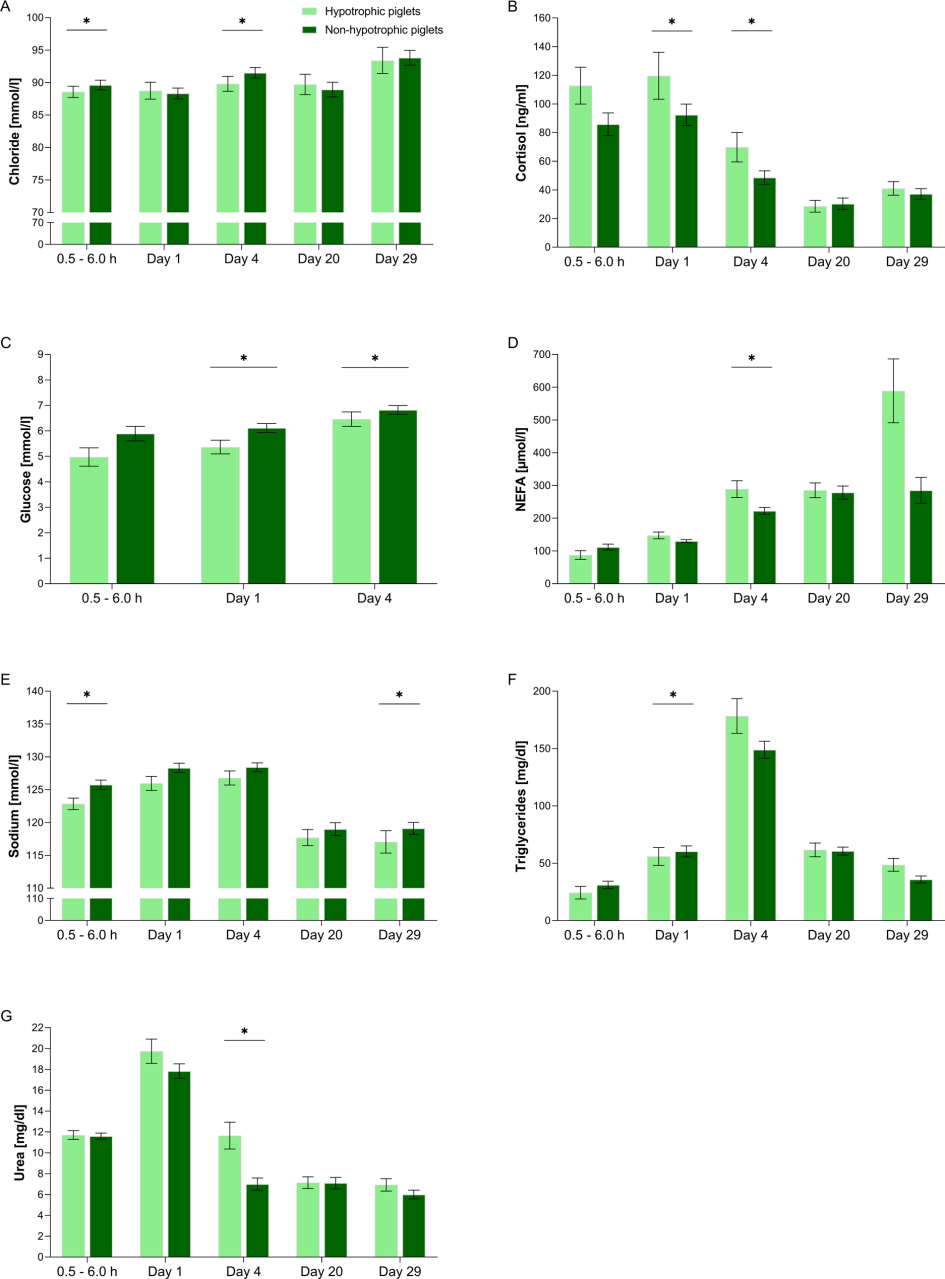
Effect of birth weight class on plasma metabolites in neonatal and suckling piglets. Plasma metabolites exhibiting significant differences for specific time points comprised (**A**) chloride, (**B**), cortisol, (**C**) glucose, (**D**) NEFA (non-esterified fatty acids), (**E**) sodium, (**F**) triglycerides, and (**G**) blood urea nitrogen. Samples were taken at 0.5–6.0 h, day 1, day 4, day 20, and day 29. Significant effects (*P* < 0.05) are indicated by asterisks

As shown in Fig. 3, hypotrophic piglets had lower plasma glucose concentrations than their non-hypotrophic littermates on day 1 (*P* = 0.006) and day 4 (*P* = 0.009). NEFA concentrations were 30% higher in hypotrophic piglets than in non-hypotrophic piglets on day 4 (*P* = 0.023). Plasma triglyceride levels in hypotrophic piglets were lower than in non-hypotrophic piglets on day 1 (*P* = 0.035). Plasma urea levels of hypotrophic piglets were 66% elevated compared to non-hypotrophic piglets on day 4 (*P* = 0.007). Cortisol levels were higher in hypotrophic piglets than non-hypotrophic littermates at day 1 (*P* = 0.013) and day 4 (*P* = 0.011). Sodium levels were lower in hypotrophic piglets compared to non-hypotrophic piglets at 0.5–6.0 h (*P* = 0.009) and day 29 (*P* = 0.005), and chloride levels were lower at 0.5–6.0 h (*P* = 0.032) and on day 4 (*P* = 0.022; Additional Table S3; Additional Table S4).

Notable time effects were detected for total protein and triglycerides. The total protein content increased significantly between samplings at 0.5–6.0 h and day 1 (*P* < 0.001). Plasma triglycerides increased between day 1 and day 4 (*P* < 0.001) and subsequently decreased until day 20 (*P* < 0.001).

## Discussion

The objective of this study was to investigate the potential influence of farrowing induction and the farrowing environment, i.e. pens or crates, on piglet vitality. For this purpose, general farrowing characteristics were determined and serial analyses of growth and plasma metabolites were performed in piglets of different birth weight classes.

### Characteristics of farrowing

Since the duration of farrowing has been shown to influence piglet vitality, particularly regarding the level of hypoxia [30], it is notable that in this experimental setting with 18 litters, neither parturition induction nor farrowing environment affected farrowing duration or intervals between births. This finding is consistent with previous studies, where Boonraungord et al. [15] could not detect an effect of parturition induction and Pedersen et al. [23] could not determine an influence of the farrowing environment on the inter-farrowing intervals and farrowing duration. However, other studies indicated a shorter farrowing duration due to parturition induction [31, 32]. The relatively high stillbirth rate in the induced parturitions from farrowing pens is noteworthy. The reasons for this cannot be attributed to farrowing duration, dystocia rate, sow conditions, and management practices (e.g. ambient temperature), as these did not show any abnormalities. Vitality immediately after birth is critical for survival, as it impacts the time to first suckling, with shorter times indicating stronger and more vital piglets. This might be reflected in the numerically lower pre-weaning mortality of spontaneous parturitions compared to induced parturitions in pens. Additionally, Boonraungrod et al. [15] reported a detrimental impact of carbetocin on the ability of newborn piglets to suckle within the initial 24-hour postnatal period. Furthermore, carbetocin may reduce the quantity of colostrum produced [15]. In addition to the adverse effects described, in the current study the time to first suckling was also significantly prolonged by parturition induction with PGF_2α_ and carbetocin compared to spontaneous parturitions.

All piglets experienced a drop in body temperature at birth. However, spontaneously born piglets appeared to regulate their body temperature more rapidly, as evidenced by higher rectal temperatures at day 1 (24 h) of life compared to piglets from induced parturitions. The more effective adaptation of thermoregulation indicates a higher vitality, which could also be expressed in a shorter time to first suckling. This finding aligns with the results of Boonraungrod et al. [15], who also observed higher rectal temperatures in spontaneously born piglets compared to those born via induced delivery at 24 h *postpartum*. There is a positive correlation between birth weight and rectal temperature shortly after birth, likely due to the less favourable surface-to-volume ratio in hypotrophic piglets [33]. However, if these piglets can successfully regulate their body temperature by day 1 of age, they are more likely to survive the suckling period. The pre-weaning mortality rates were not affected by the farrowing environment in this experimental setting with 18 litters, consistent with the findings of Kilbride et al. [11].

### Body weight development

The impact of parturition induction on weaning weight is inconsistent across studies [18, 34, 35], suggesting additional interacting factors such as nutrition, fat mobilization, microbial colonization, or health status (e.g. *postpartum* dysgalactia syndrome prevalence) during the suckling period in sows and piglets. In our study, there was no significant association between body weight and farrowing environment, except at day 20. In the same way, there was no significant relationship with the proportion of hypo-, eu-, or hypertrophic piglets, which is consistent with previous research findings [36]. This indicates that nutrient intake by piglets is maintained regardless of the sow’s freedom of movement. However, piglets with low birth weight were unable to compensate for the weight difference until shortly after weaning, regardless of parturition induction and farrowing environment, a phenomenon observed already in other studies [37–39].

### Effect of parturition induction

Approximately ten hours after birth, piglets’ endogenous glycogen reserves are depleted [40]. To meet their nutritional requirements, piglets must therefore engage in gluconeogenesis. In this study, piglets born spontaneously exhibited lower plasma concentrations of glucose and lactate during the initial four-day period compared to piglets born via induced delivery. The lower plasma glucose values suggest a higher glucose turnover, while the lower lactate values suggest the possibility of increased gluconeogenesis. Lower plasma glucose levels in piglets from non-induced parturitions were similarly observed by Sanchez-Aparicio et al. [41] when measured directly after birth. In the current study, the differences in the first measurements *postnatum* (0.5–6.0 h) were numerical but not statistically significant. One possible explanation could be that the first intake of colostrum before sampling masked these effects. In addition to carbohydrates, energy is also obtained from lipids via mobilization of energy reserves. The higher NEFA values in spontaneously born piglets on day 4 might support the assumption for a higher energy requirement compared to piglets from induced births. In contrast, the results could be due to a potentially increased intake of colostrum in pigs from non-induced sows, as these pigs have suckled earlier and a higher intake is to be expected. The observed overall increase in total protein concentration on day 1, along with the survival of the suckling period, provides evidence that the piglets had sufficient colostrum ingestion and absorption. Indeed, most colostrum proteins are highly digestible [42] and IgG serves as major component of colostral proteins ultimately elevating plasma total protein concentration in neonates [3, 43]. The total protein levels were found to be higher in spontaneously born piglets on day 4 and 20, which might indicate differences in protein turnover. The elevated blood urea nitrogen levels observed in spontaneously born piglets on day 20 may be attributed to an assumed increased protein turnover. It is similarly conceivable that piglets from spontaneous parturitions employed less protein for energy production during the neonatal adaptation phase, resulting in lower ammonia levels and higher total protein concentrations on day 1. In fact, metabolic and endocrine drivers do not necessarily peak at the same time due to differences in responsiveness to external cues (e.g., suckling time) and intrinsic circadian regulation. Lower ammonia levels have also been observed when protein is used to build lean muscle mass [44]. Increased plasma uric acid concentrations shortly after birth have been associated with hypoxia [45]. Piglets from spontaneous parturitions also have higher plasma uric acid levels than piglets born by caesarean Sect. [46]. However, the piglets from spontaneous parturitions in the current study are unlikely to have been hypoxic, as the lactate levels do not differ from piglets from induced parturitions. This is supported by the study by Wehrend et al. [47], who did not find any difference in the blood pH level of piglets from induced and spontaneous parturition immediately after delivery. As the kidneys are not fully developed [48], there may be certain difficulties with both sodium reabsorption and excretion [49]. The lower sodium levels of the spontaneous group during the neonatal adaptation phase may therefore be due to adaptation processes. The differences in creatinine levels between piglets born from spontaneous parturition and those from induced parturition can be attributed to metabolic activity and renal clearance. Piglets born through spontaneous parturition may experience more active muscle contractions during delivery, leading to higher creatinine production initially. By day 1, induced piglets might catch up as they begin normal postnatal muscle activity. Lower haptoglobin concentrations were found in piglets born by spontaneous delivery compared to induced parturitions. Haptoglobin is absorbed with the colostrum of piglets and stimulates its endogenous genesis [50, 51]. There should be no significant response to external stressors at this age [52] and an increase in plasma haptoglobin concentration in the first week of life has been interpreted as a strategy for surviving this critical life phase [53]. In this context, haptoglobin has been suggested to play a crucial role for haemoglobin recycling and iron conservation as reviewed elsewhere [54, 55].

### Impact of the farrowing environment

Results revealed differences in lactate concentrations due to the applied farrowing environment, indicating increased gluconeogenesis in pen-born piglets (day 1). The latter is consistent with findings from Devillers et al. [3], who concluded a higher energy requirement for pen-born piglets during the neonatal period. The assumption for a high energy requirement might be also supported by the lower plasma glucose values in pen-housed piglets compared to those with a crated dam on day 1 in this study. In contrast, Nowland et al. [56] reported higher blood glucose levels one day *postpartum* in piglets born in farrowing pens compared to those born in gestation crates, which they attributed to higher energy intake. However, neither the two studies cited, nor the current study, state how much time elapsed between the last suckling and blood sampling. It is known from human medicine that the plasma inositol level in newborn infants is high, e.g. due to neural development, and rapidly declines in the first days of life to reach baseline levels [57]. The lower inositol levels in piglets in pens compared to those with a crated dam might suggest a slightly higher efficiency in renal elimination. There are also differences in protein and nitrogen metabolism depending on whether the mothers are kept in a pen or a crate. In the first few days, the piglets use proteins for energy synthesis, whereby urea is formed [58]. Higher blood urea nitrogen concentrations in piglets from the pen group indicate a higher protein turnover in the first hours of life. In addition, an assumed higher physical activity of piglets from sows kept in pens not only leads to higher energy requirements from carbohydrates, fat and protein metabolism, but also to higher creatinine concentrations [59], similar to our results.

### Effect of birth weight class

Hypotrophic piglets have a relatively higher energy requirement per kg body weight due to their less favourable surface-to-volume ratio compared to their normal-weight counterparts [33]. This was reflected in the lower glucose concentrations on day 1 and day 4. In addition, hypotrophic piglets may have absorbed less nutrients [60], as the milk composition varies between the individual teats [61] and there is also teat competition in the first few days of life [62, 63]. The individual teat order was not recorded in this study. Furthermore, the hypotrophic piglets exhibited low glucose concentrations which might have led to an increased glucose mobilization via counter-regulatory hormones such as cortisol. Cortisol also has a catabolic effect, which results in higher energy consumption and the mobilization of energy reserves. However, in another study no marked differences in cortisol response were observed during hypoglycemic clamps in pigs [64]. Additionally, cortisol is known to play a crucial role in promoting surfactant production and lung maturation in neonatal pigs, and low birth weight piglets may have different cortisol dynamics compared to their normal birth weight counterparts. Colostrum intake is significantly dependent of the birth weight class [60]. Therefore, the low birth weight piglets have a smaller amount of nutrients at their disposal. In catabolic conditions, lipids are also mobilized. This may be a reason for higher NEFA levels on day 4. At the same time, there is a high demand for energy, while low endogenous energy reserves are available in hypotrophic piglets [65]. In addition, differences in the maturation state of pancreas are conceivable, which might lead to an accumulation of NEFA in the plasma [66].

In this study, the selected hypotrophic piglets appeared to be sufficiently developed to survive, as the fructose levels did not differ from those of the non-hypotrophic piglets [1, 67, 68]. Higher plasma concentrations of blood urea nitrogen as a product of protein deamination occur in hypotrophic piglets if the proteins are used to cover energy requirements rather than for the accretion of lean muscle tissue [69, 70]. Therefore, Decaluwé et al. [71] also reports a negative correlation between plasma urea levels and daily weight gain.

Hypotrophic piglets had lower plasma sodium levels than the non-hypotrophic piglets a few hours *postnatum*. As blood electrolytes are tightly regulated, results indicate that kidney function is not yet developed, leading to a reduced ability to regulate and retain sodium. The sodium levels are also lower in the hypotrophic piglets than in the non-hypotrophic piglets one day after weaning. This is probably associated with the acid-base balance [1]. This underlines the importance of appropriate nutritional and management strategies to promote the health and growth of low weight piglets during the neonatal adaptation period and beyond.

Notably, this study was carried out on a limited number of plasma sampling time points to cover important adaptation processes of neonates and suckling piglets. This may have resulted in certain dynamic changes in metabolite profiles during neonatal adaptation and lactation remaining unrecognized.

## Methods

### Farrowing environment, parturition induction, and management

**Fig. 4:**
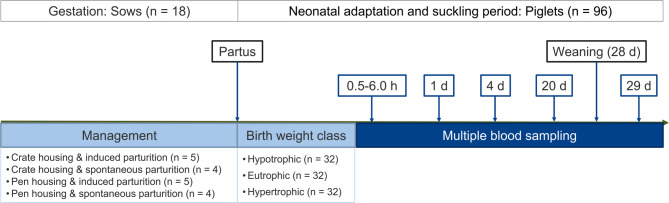
Experimental design to evaluate piglet´s metabolism due to farrowing environment, parturition induction, and birth weight class. Multiparous German Landrace (GL) sows were subjected to farrow in two different farrowing environments, i.e., crate or pen (*n* = 9 each). A subset of sows (crate: *n* = 5, pen: *n* = 5) underwent induced parturition, while another subset (crate: *n* = 4, pen: *n* = 4) experienced spontaneous farrowing. In total, *n* = 283 piglets were classified according to birth weight as hypotrophic (800–1100 g), eutrophic (> 1100–1500 g), and hypertrophic (> 1500 g). Of these, *n* = 96 piglets were selected for repeated blood analyses during the entire suckling period. Piglets from induced parturitions have also been implemented in a companion study [29]

The study was based on 96 extensively examined piglets from a total of 18 German Landrace sows (GL) of parities two to six as part of a larger study [29]. All animals were owned by the Research Institute of Farm Animal Biology (FBN) and were reared and housed in its Experimental Swine Facilities. All sows were artificially inseminated and kept in groups until they were moved to farrowing units at 105 days *post*-*conceptionem* (dpc). The sows were allocated randomly to four treatment groups based on their farrowing environment and nature of parturition onset: (i) Crate-housed with induced parturition, (ii) crate-housed with spontaneous parturition, (iii) pen-housed with induced parturition, and (iv) pen-housed with spontaneous parturition (Fig. 4). In detail, sows were placed in crates (*n* = 9) or pens (*n* = 9) on day 114 of gestation. On day 114 of gestation, farrowing induction was performed on five sows in each of the two farrowing environments (crate: *n* = 5; pen: *n* = 5). Parturition was induced with a standard regimen of prostaglandin F_2α_ (PGF) and carbetocin. Specifically, 0.175 mg cloprostenol (2 ml PGF Veyx forte^®^; Veyx, Schwarzenborn, Germany) was injected intramuscularly on gestation day 114. Furthermore, the respective sows received 70 µg carbetocin (1 ml Depotocin^®^; Veyx, Schwarzenborn, Germany) intramuscularly on gestation day 115 (24 h after PGF). The piglets obtained from the induced parturitions were previously analysed [29] and now serve as a reference group in the present study. In addition, two experimental groups did not receive hormonal parturition induction to represent spontaneous, non-induced birth conditions (crate: *n* = 4; pen: *n* = 4).

Both farrowing environments had a total floor area of 6.5 m². In the crate system, the crate was closed for five consecutive days starting one day before expected farrowing. The area available to the sows was limited to 180 × 60–70 cm in the crate system. In the pen system, the sows had access to the entire 6.5 m² area (325 × 200 cm), except for the creep area. In both farrowing environments, a heating plate (76 × 60 cm) and a heat lamp (creep area temperature 30 °C) were provided for the piglets in the creep area. The temperature in the farrowing pens was set at 22 °C and computer-controlled along with the ventilation.

At 109 dpc, the sows were transitioned from a commercial gestation diet (12.2 MJ of metabolizable energy (ME), 3.2 kg/d; Additional Table S5) to a commercial lactation diet (13.2 ME, 3.2 kg/d), which was subsequently reduced to 2 kg/d at 112 dpc. Following farrowing, the quantity was augmented to a maximum of 8 kg at mid-lactation, contingent on individual requirements. Water was available *ad libitum* to all animals via one drinker per farrowing unit.

### Supervision of sows and piglets

The peripartal period was monitored personally for all sows. The parturition was defined as the time between the first and the last piglet being born. In the case that the inter-farrowing interval exceeded 60 min, manual obstetrics was performed without the administration of hormones.

The number of live-born piglets, stillborn piglets, and mummified piglets was recorded per litter. The individual time of parturition was recorded for each piglet and inter-farrowing intervals were calculated. Piglet vitality was scored based on Zaleski and Hacker (1993) [72]. For breathing, skin colour, standing attempts, and meconium staining, scores with the levels 0, 1, or 2 were assigned for each parameter. This resulted in vitality scores that ranged between 0 (death) and 8 (best) as described previously [29]. Due to visual inspection, the time to first suckling was recorded for each neonatal piglet. The individual body weight of all piglets was determined after their first suckling event using a digital scale (Kern & Sohn, Balingen, Germany). Body weight of the selected piglets was also determined on day 1 (24 h), day 4, day 20 and day 29. The rectal body temperature was measured (Microlife VT 1831; Mikrolife, Effretikon, Switzerland) after colostrum intake and on day 1 (24 h). Piglet mortality was recorded daily during suckling period.

The management of neonatal and suckling piglets followed the procedure described previously [29]. In brief, piglets were marked with individual tattoo numbers on the first day of life. The piglets received an oral iron and vitamin application (PUCORAL^®^ FerroPlus, Pulte GmbH & Co. KG, Germany) on the first day of life (1 ml per piglet) and an iron-injection (Ursoferran^®^ 100 mg/ml, Serumwerk Bernburg, Germany) on day 14 of life (2 ml per piglet). The piglets were fed a pre-starter (Hakra-Immuno-G, 15.2 MJ ME/kg, 18.8% CP, Una Hakra) *ad libitum* via an automatic feeder from 14 days of life. They were vaccinated against *Lawsonia intracellularis* (ENTERISOL^®^ ILEITIS, Boehringer Ingelheim Vetmedica GmbH, Germany) at three weeks of age. Piglets from spontaneous parturitions were weaned at 27.8 ± 0.5 days of life (induced parturitions: 28 days of life).

### Piglet selection

Results on a subgroup of piglets, i.e., induced parturitions, have been presented previously [29] and now serve as a reference group in the present study. After first suckling, the piglets were classified by body weight (*n* = 283). The applied weight classes were based on the stock percentiles of 13,755 neonatal piglets from the FBN experimental station (FBN, unpublished data). Piglets with initial body weight < 1100 g were defined as hypotrophic (lowest quartile), from 1100 to 1500 g as eutrophic, and > 1500 g as hypertrophic (highest quartile). To minimize effects from intrauterine growth retardation (IUGR), a cut-off was applied at a birth weight < 800 g for selecting piglets sampled over the suckling period, i.e., piglets weighing < 800 g were excluded from the metabolite profiling. In each of the four experimental sow groups, eight piglets per body weight class were selected randomly from 4 to 5 litters (*n* = 96) for monitoring and repeated blood sampling throughout the suckling period (Fig. 4).

### Repeated blood collection of piglets

Blood samples were collected from the 96 selected piglets (2 induction protocols × 2 farrowing environments × 3 birth weight classes × 8 biological replicates) via the vena jugularis after the first suckling between 0.5 and 6.0 h of age, with precise time of collection recorded. Additional blood samples were taken at day 1 (24 h) and day 4 to cover the neonatal adaptation phase (Fig. 4). The blood samples taken at day 20 and day 29 were supposed to reflect the effects of organ maturation and weaning. Blood samples were collected using K-EDTA tubes (Sarstedt, Nürnbrecht, Germany). EDTA samples were placed on ice and centrifuged (4 °C, 3500 rpm, 15 min). Retrieved plasma was stored at -80 °C until analysis.

### Physiologic parameters, hormones and metabolites

As a follow up to our recent publication [29], a number of plasma metabolites was analysed using commercially available kits, including total protein, albumin, blood urea nitrogen, creatinine, ammonia, triglycerides, uric acid, sodium, and chloride (FUJI Dri-Chem 4000i, FujiFilm, Minato, Japan). Plasma concentrations of fructose, glucose, inositol, and lactate were measured by HPLC (Agilent, Waldbronn, Germany) as previously described [73]. In brief, these metabolites were determined after protein precipitation with 30 µl plasma and 20 µl 1.5 M perchloric acid and neutralization with 10 µl 2 M K_2_CO_3_ and centrifuged (4 °C, 50.000 g rpm, 20 min). Plasma levels of non-esterified fatty acids (NEFA) were determined with a commercial device (ABX Pentra C400, HORIBA Medical, Montpellier, France) with enzyme-based kits (WAKO Chemicals GmbH, Neuss, Germany). Plasma haptoglobin (cat.-no. HAPT-9, Life Diagnostics, Inc., West Chester, Pennsylvania, USA), cortisol (cat.-no. EIA-1887, DRG Diagnostics GmbH, Marburg, Germany), insulin (cat.-no. EIA-4747, DRG Diagnostics GmbH), and total triiodothyronine (cat.-no. EIA-4569, DRG Diagnostics GmbH) were measured in duplicate via ELISAs according to the manufacturer’s protocol.

An adequate number of animals was used (effect size: Cohen’s f = 0.3; power: 80%; maximum error: 5%), and only limited volumes of blood were repeatedly sampled. Consequently, not all parameters could be measured at every time point due to the limited availability of plasma. An overview of the respective plasma parameters determined per time point is shown in Additional Table S2.

### Statistical analyses

Regarding farrowing traits (e.g. farrowing duration, litter size), the litter was the experimental unit. Data was analysed using a two-way ANOVA taking into account the farrowing environment and the parturition induction as fixed effects (R language v4.2.2; R package ‘car’, v3.1-1; R foundation for statistical computing, Vienna, Austria). The Tukey post-hoc test was used for pairwise comparisons between experimental piglet groups. Based on previously published results of the project [29], eutrophic and hypertrophic piglets were grouped together as non-hypotrophic piglets for the statistical analyses. A chi-square test was performed to test whether there is an influence of birth weight class on pre-weaning mortality.

The individual piglet was the experimental unit for analyses of the plasma metabolites. A rank-based inverse normal transformation was applied as raw data showed deviation from normal distribution (R package ‘RNOmni’, v1.0.1.2). In a repeated measurements approach, the individual animal was considered and the effects of parturition induction, farrowing environment, birth weight class, and time as fixed effects as well as the respective two-way and three-way interactions were analysed (R package ‘stats’).

Additionally, observations at each individual time point were evaluated via a linear model with parturition induction, farrowing environment, and birth weight class as fixed effects as well as their respective two-way and three-way interactions (R package ‘stats’). In order to evaluate possible time effects on the total plasma protein (0.5–6.0 h vs. day 1) and plasma triglyceride levels (day 1 vs. day 4 vs. day 20), the linear model included the effect of sampling time point. The difference between age at sampling and first colostrum intake, showing the individual duration for digestion of macronutrients, was considered as covariate for calculations related to the initial sampling time point (0.5–6.0 h). The pairwise comparison of the mean values between experimental groups was carried out using the Tukey post-hoc test. The significance level was set at *P* < 0.05. All results were presented as mean ± SEM.

## Conclusion

Compared to piglets from induced parturitions, piglets from spontaneous parturitions appear to be more mature and utilise nutrients more effectively. Metabolic differences between piglets in pens and piglets with a crated dam indicate changes in energy metabolism and physical activity. Hypotrophic piglets showed clear metabolic changes compared to heavier littermates in order to mobilise energy resources in the first few days of life. Sow management, including farrowing environment and the nature of parturition onset, plays a crucial role in improving piglet metabolism and should be individualised. By challenging the routine use of parturition induction and investing in more labor-intensive, individualized management practices, pig farmers could improve both piglet maturity and vitality.

## Electronic supplementary material

Below is the link to the electronic supplementary material.

Supplementary Material 1

Supplementary Material 2

Supplementary Material 3

Supplementary Material 4

Supplementary Material 5

## Data Availability

Part of the data generated or analysed is contained in this published article [31]. The data that support the study findings are available from the authors upon request.

## Abbreviations

Dpc: Days post-conceptionem
I: Parturition induction
F: Farrowing environment
ME: Metabolizable energy
