# Supplementary material for: Impact of parturition induction, farrowing environment and birth weight class on endocrine and metabolic plasma parameters related to piglet vitality
Source: BMC Vet Res. 2025 Jun 7;21:406. doi: 10.1186/s12917-025-04845-2 (PMC12144723; doi:10.1186/s12917-025-04845-2)
Supplement: Supplementary file 1 — Supplementary Material 1 [file 12917_2025_4845_MOESM1_ESM.pdf]

Impact of partus induction, housing system and birth weight class on endocrine and metabolic plasma parameters related to piglet vitality

H Lickfett[1,2], M Oster[1], A Vernunft[1], H Reyer[1], E Muráni[1], S Görs[1], CC Metges[1], H Bostedt[2], K Wimmers[1,3,*]

[1]Research Institute for Farm Animal Biology (FBN), 18196 Dummerstorf, Germany;

[2]Veterinary Clinic for Reproductive Medicine and Neonatology, Justus-Liebig-University Gießen, 35392 Gießen, Germany;

[3]Chair of Animal Breeding and Genetics, Faculty of Agricultural and Environmental Sciences, University Rostock, 18059 Rostock, Germany;

*Correspondence: Email: wimmers@fbn-dummerstorf.de; Tel.: +49-38208-68-600;

Supplemental Table S1: Means and SEM of selected plasma metabolites in neonates and suckling piglets.

Time point	Induced parturition				Spontaneous parturition			
	Crate		Pen		Crate		Pen	
	800-1100 g	>1100 g	800-1100 g	>1100 g	800-1100 g	>1100 g	800-1100 g	>1100 g
Albumin [g/dl]								
0.5-6.0 h	0.99 ± 0.05	0.95 ± 0.11	1.28 ± 0.07	1.03 ± 0.12	0.99 ± 0.07	0.95 ± 0.07	1.01 ± 0.05	1.10 ± 0.13
1 d	1.99 ± 0.13	1.74 ± 0.14	1.78 ± 0.08	1.59 ± 0.12	1.74 ± 0.06	1.74 ± 0.07	1.96 ± 0.10	1.90 ± 0.10
4 d	2.23 ± 0.11	2.09 ± 0.08	2.16 ± 0.06	2.11 ± 0.11	2.19 ± 0.05	2.19 ± 0.11	2.27 ± 0.10	2.11 ± 0.11
20 d	3.71 ± 0.16	4.20 ± 0.20	3.97 ± 0.18	3.87 ± 0.12	3.81 ± 0.12	3.83 ± 0.17	3.58 ± 0.16	3.86 ± 0.11
29 d*	3.76 ± 0.16	3.80 ± 0.30	3.75 ± 0.12	4.23 ± 0.39	3.77 ± 0.09	3.70 ± 0.13	3.48 ± 0.11	3.61 ± 0.15
Chloride [mmol/l]								
0.5-6.0 h	89.87 ± 1.74	86.13 ± 1.98	87.56 ± 1.53	89.00 ± 1.61	90.20 ± 1.45	90.88 ± 1.14	90.88 ± 1.24	88.38 ± 1.79
1 d	90.94 ± 1.98	88.14 ± 2.54	87.82 ± 1.97	91.63 ±3.45	86.38 ± 1.44	89.00 ± 2.10	88.19 ± 0.84	86.13 ± 2.22
4 d	92.53 ± 1.70	86.63 ± 2.35	88.71± 1.15	91.71 ± 2.56	92.00 ± 1.47	92.25 ± 2.08	93.00 ± 1.96	88.88 ± 2.10
20 d	87.27± 2.99	89.83 ± 4.98	86.18 ± 1.87	91.00 ± 2.45	90.73 ± 2.40	90.38 ± 2.70	91.69 ± 1.58	88.00 ± 3.01
29 d*	93.33 ± 2.78	84.60 ± 4.59	93.00 ± 2.04	96.83 ± 5.52	94.13 ± 1.64	97.63 ± 3.26	94.81 ± 2.55	92.13 ± 2.37
Creatinine [mg/dl]								
0.5-6.0 h	1.39 ± 0.12	1.45 ± 0.22	1.78 ± 0.11	2.13 ± 0.28	1.78 ± 0.13	1.66 ± 0.14	1.55 ± 0.08	1.48 ± 0.12
1 d	0.74 ± 0.04	0.78 ± 0.07	1.04 ± 0.04	1.15 ± 0.06	1.00 ± 0.05	0.92 ± 0.06	0.89 ± 0.05	1.01 ± 0.11
4 d	0.63 ± 0.02	0.56 ± 0.02	0.90 ± 0.03	0.91 ± 0.05	0.83 ± 0.05	0.74 ± 0.06	0.73 ± 0.03	0.67 ± 0.05
20 d	0.80 ± 0.05	0.69 ± 0.09	0.89 ± 0.03	1.00 ± 0.08	0.97 ± 0.06	0.89 v0.08	1.06 ± 0.09	1.24 ± 0.17
29 d*	0.80 ± 0.03	0.68 ± 0.08	1.14 ± 0.02	1.23 ± 0.12	0.97 ± 0.09	0.99 ± 0.10	1.25 ± 0.06	1.22 ± 0.05
Glucose [mmol/l]								
0.5-6.0 h	6.70 ± 0.75	5.94 ± 0.50	5.93 ± 0.54	3.74 ± 0.45	5.88 ± 0.46	5.74 ± 0.87	5.06 ± 0.48	4.18 ± 0.66
1 d	7.01 ± 0.47	5.25 ± 0.76	5.65 ± 0.31	4.74 ± 0.60	5.86 ± 0.27	6.24 ± 0.29	5.94 ± 0.24	5.22 ± 0.31
4 d	7.94 ± 0.22	6.41 ± 0.99	6.67 ± 0.43	6.58 ± 0.22	6.73 ± 0.29	6.63 ± 0.47	6.04 ± 0.21	6.25 ± 0.27
Haptoglobin [µg/ml]								
0.5-6.0 h	43.70 ± 11.03	17.54 ± 7.35	80.47 ± 15.48	36.87 ± 20.89	32.69 ± 8.40	30.02 ± 13.04	31.11 ± 10.31	52.20 ± 18.78
1 d	116.25 ± 8.79	89.58 ± 15.87	114.43 ± 20.75	174.20 ± 40.49	86.38 ± 9.24	118.86 ± 11.63	118.97 ± 13.54	114.73 ± 14.52
4 d	162.85 36.40	206.47 ± 76.58	107.17 ± 40.52	227.78 ± 89.62	197.45 ± 37.19	96.70 ± 35.11	152.55 ± 44.15	160.28 ± 50.27
20 d	421.11 ± 186.66	736.79 ± 392.66	213.85 ± 112.25	75.24 ± 53.01	180.60 ± 43.93	165.33 ± 81.96	118.00 ± 31.45	115.57 ± 39.12
29 d*	454.16 ± 184.41	802.27 ± 446.21	395.28 ± 73.93	448.37 ± 162.09	363.16 ± 46.31	445.65 ± 42.24	417.09 ± 36.72	435.63 ± 38.67
Inositol [mmol/l]								
0.5-6.0 h	4.39 ± 0.48	5.45 ± 0.44	3.34 ± 0.39	6.09 ± 0.59	5.05 ± 0.74	5.86 ± 0.72	3.28 ± 0.39	5.40 ± 0.87

1 d	3.15 ± 0.32	3.30 ± 0.54	2.23 ± 0.26	4.13 ± 0.42	2.65 ± 0.35	3.06 ± 0.40	2.67 ± 0.26	3.53 ± 0.48
Lactate [mmol/l]								
0.5-6.0 h	3.98 ± 0.40	4.34 ± 0.54	4.27 ± 0.32	5.03 ± 0.69	4.79 ± 0.98	4.12 ± 0.59	4.19 ± 0.37	3.58 ± 0.28
1 d	6.31 ± 0.68	6.75 ± 0.83	4.54 ± 0.21	5.17 ± 0.30	4.92 ± 0.48	4.78 ± 0.41	5.23 ± 0.65	3.89 ± 0.59
4 d	6.46 ± 0.74	7.44 ± 0.84	6.68 ± 0.61	5.39 ± 0.89	4.57 ± 0.42	3.70 ± 0.34	5.08 ± 0.53	4.11 ± 0.41
Sodium [mmol/l]								
0.5-6.0 h	127.86 ± 1.92	121.33 ± 1.78	128.17 ± 0.93	123.50 ± 1.85	124.00 ± 0.96	125.50 ± 0.91	123.63 ± 1.19	121.00 ± 1.93
1 d	132.67 ± 2.04	128.33 ± 1.71	128.54 ± 1.09	131.57 ± 1.39	126.94 ± 0.79	122.50 ± 0.82	125.44 ± 0.79	122.75 ± 2.24
4 d	131.08 ± 1.79	128.38 ± 2.13	130.82 ± 1.03	127.14 ± 1.94	126.38 ± 1.13	128.63 ± 2.46	125.88 ± 1.17	123.00 ± 1.65
20 d	118.83 ± 2.74	123.50 ± 3.71	122.67 ± 1.39	115.75 ± 4.44	120.50 ± 1.27	116.63 ± 0.92	115.53 ± 1.73	116.71 ± 1.87
29 d*	122.36 ± 2.48	110.00 ± 5.51	121.64 ± 1.21	125.50 ± 3.84	118.79 ± 1.45	117.29 ± 1.69	114.75 ± 1.27	115.00 ± 2.60
Total protein [g/dl]								
0.5-6.0 h	2.48 ± 0.10	2.38 ± 0.20	3.14 ± 0.20	2.48 ± 0.26	2.55 ± 0.14	2.48 ± 0.20	2.57 ± 0.11	2.78 ± 0.29
1 d	4.99 ± 0.23	4.43 ± 0.42	4.64 ± 0.17	4.16 ± 0.38	5.18 ± 0.19	5.04 ± 0.22	5.63 ± 0.18	5.58 ± 0.24
4 d	4.54 ± 0.17	4.30 ± 0.21	4.44 ± 0.10	4.36 ± 0.24	5.10 ± 0.11	4.81 ± 0.21	5.26 ± 0.14	4.99 ± 0.17
20 d	4.33 ± 0.13	4.72 ± 0.16	4.76 ± 0.35	4.18 ± 0.12	4.80 ± 0.15	4.51 ± 0.18	4.50 ± 0.11	4.66 ± 0.13
29 d*	4.49 ± 0.14	4.42 ± 0.25	4.33 ± 0.11	4.65 ± 0.63	4.67 ± 0.11	4.43 ± 0.10	4.31 ± 0.13	4.41 ± 0.11
Triglyceride [mg/dl]								
0.5-6.0 h	40.47 ± 6.45	18.86 ± 8.70	34.44 ± 6.22	42.17 ± 19.63	29.87 ± 5.71	16.25 ± 6.95	20.19 ± 6.93	23.88 ± 8.23
1 d	62.81 ± 10.53	29.86 ± 6.92	48.35 ± 6.71	53.13 ± 19.82	73.25 ± 9.68	78.75 ± 16.83	57.75 ± 10.93	59.00 ± 11.34
4 d	153.67 ± 10.97	199.00 ± 35.68	168.65 ± 20.38	165.29 ± 11.41	138.69 ± 9.79	164.38 ± 40.74	133.88 ± 13.95	183.13 ± 26.03
20 d	62.00 ± 8.47	54.00 ± 10.91	61.88 ± 6.58	79.67 ± 14.07	66.73 ± 6.78	54.75 ± 9.78	52.38 ± 5.79	60.88 ± 13.31
29 d*	38.43 ± 7.67	57.80 ± 21.32	30.63 ± 3.57	48.00 ± 10.90	37.53 ± 6.35	54.88 ± 6.28	37.38 ± 6.53	37.13 ± 9.61
Urea [mg/dl]								
0.5-6.0 h	10.78 ± 0.28	11.41 ± 0.72	12.94 ± 0.65	12.73 ± 1.32	12.13 ± 0.37	12.33 ± 0.56	10.56 ± 0.75	10.63 ± 0.75
1 d	18.68 ± 1.62	22.13 ± 2.37	19.36 ± 1.43	19.39 ± 2.62	17.31 ± 1.34	19.04 ± 1.17	15.89 ± 1.05	18.73 ± 2.98
4 d	6.28 ± 0.66	12.16 ± 1.79	7.21 ± 1.09	11.24 ± 1.73	7.49 ± 1.50	15.51 ± 3.95	6.96 ± 1.34	7.61 ± 1.43
20 d	5.21 ± 0.66	7.45 ± 0.80	6.25 ± 0.63	5.15 ± 0.95	9.11 ± 0.86	8.48 ± 1.31	7.88 ± 1.69	7.08 ± 0.95
29 d*	5.13 ± 0.84	6.88 ± 1.33	5.52 ± 0.65	7.12 ± 1.04	6.39 ± 0.89	6.29 ± 1.02	6.93 ± 0.93	7.46 ± 1.43

* post-weaning;
