# Supplementary material for: Impact of parturition induction, farrowing environment and birth weight class on endocrine and metabolic plasma parameters related to piglet vitality
Source: BMC Vet Res. 2025 Jun 7;21:406. doi: 10.1186/s12917-025-04845-2 (PMC12144723; doi:10.1186/s12917-025-04845-2)
Supplement: Supplementary file 2 — Supplementary Material 2 [file 12917_2025_4845_MOESM2_ESM.pdf]

Impact of partus induction, housing system and birth weight class on endocrine and metabolic plasma parameters related to piglet vitality

H Lickfett[1,2], M Oster[1], A Vernunft[1], H Reyer[1], E Muráni[1], S Görs[1], CC Metges[1], H Bostedt[2], K Wimmers[1,3,*]

[1]Research Institute for Farm Animal Biology (FBN), 18196 Dummerstorf, Germany;

[2]Veterinary Clinic for Reproductive Medicine and Neonatology, Justus-Liebig-University Gießen, 35392 Gießen, Germany;

[3]Chair of Animal Breeding and Genetics, Faculty of Agricultural and Environmental Sciences, University Rostock, 18059 Rostock, Germany;

*Correspondence: Email: wimmers@fbn-dummerstorf.de; Tel.: +49-38208-68-600;

Supplemental Table S5. Analysed nutrient composition of the diets fed to sows during gestation and lactation and to piglets from day 14 of life.

Item[1]	Unit	Gestation diet	Lactation diet	Pre-starter diet
Crude protein	%	14.5	17.5	18.8
Crude fat	%	4.5	6.5	9.2
Crude fibre	%	7.0	5.0	2.7
Crude ash	%	5.7	6.0	4.6
Lysine	%	0.67	1.05	1.62
Methionine	%	0.23	0.32	0.62
Calcium	%	0.65	0.89	0.63
Phosphorus	%	0.54	0.63	0.54
Sodium	%	0.25	0.25	0.24
ME (pig)	MJ/kg	12.2	13.2	15.2

[1] dry matter basis; ME: metabolisable energy;

The nutritional compositions of diets were analyzed by the Agricultural Testing and Research Institute (LUFA Rostock, Germany), which is accredited as a testing laboratory and operates a quality management system in accordance with DIN EN ISO/IEC 17025 standards. Representative samples of each diet were analyzed via standard methods as described by the Association of German Agricultural Inspection and Research Institutes (methods 4.1.2, 5.1.1, 6.1.1, 7.2.1, 8.1, 10.3.2, 10.6.1).

References:

Verband Deutscher Landwirtschaftlicher Untersuchungs- und Forschungsanstalten (VDLUFA) (2007): Handbuch der Landwirtschaftlichen Versuchs- und Untersuchungsmethodik (VDLUFA-Methodenbuch), Vol. III. Die chemische Untersuchung von Futtermitteln. Darmstadt, Germany: VDLUFA-Verlag. url: https://www.methodenbuch.de/produkt/methodenbuch-band-iii-futtermittel.
