# Supplementary material for: Impact of parturition induction, farrowing environment and birth weight class on endocrine and metabolic plasma parameters related to piglet vitality
Source: BMC Vet Res. 2025 Jun 7;21:406. doi: 10.1186/s12917-025-04845-2 (PMC12144723; doi:10.1186/s12917-025-04845-2)
Supplement: Supplementary file 3 — Supplementary Material 3 [file 12917_2025_4845_MOESM3_ESM.pdf]

Impact of partus induction, housing system and birth weight class on endocrine and metabolic plasma parameters related to piglet vitality

H Lickfett[1,2], M Oster[1], A Vernunft[1], H Reyer[1], E Muráni[1], S Görs[1], CC Metges[1], H Bostedt[2], K Wimmers[1,3,*]

[1]Research Institute for Farm Animal Biology (FBN), 18196 Dummerstorf, Germany;

[2]Veterinary Clinic for Reproductive Medicine and Neonatology, Justus-Liebig-University Gießen, 35392 Gießen, Germany;

[3]Chair of Animal Breeding and Genetics, Faculty of Agricultural and Environmental Sciences, University Rostock, 18059 Rostock, Germany;

*Correspondence: Email: wimmers@fbn-dummerstorf.de; Tel.: +49-38208-68-600;

Supplemental Table S4: Plasma metabolites covering the organ maturity of neonates and suckling piglets.

Time point	Parturition induction		Farrowing environment		Birth weight class		Interactions				*P*-value time between first suckling and blood sampling
	Induced	Spontaneous	Crate	Pen	800-1100g	>1100g	Birth induction × farrowing environment	Birth induction × birth weight class	Farrowing environment × birth weight class	Birth induction × farrowing environment × birth weight class	
Albumin [g/dl]											
0.5-6.0 h	1.09 ± 0.04	1.01 ± 0.04	**0.98 ± 0.04^{A}**	**1.12 ± 0.04^{B}**	1.01 ± 0.05	1.07 ± 0.03	0.371	0.594	0.075	0.174	**<0.001**
1 d	1.81 ± 0.06	1.84 ± 0.04	1.82 ± 0.05	1.82 ± 0.05	1.74 ± 0.05	1.86 ± 0.05	**0.024**	0.365	0.949	0.849	NA
4 d	2.16 ± 0.05	2.20 ± 0.04	2.18 ± 0.05	2.18 ± 0.05	2.13 ± 0.05	2.21 ± 0.04	0.858	0.805	0.932	0.682	NA
20 d	3.90 ± 0.10	3.74 ± 0.07	3.83 ± 0.08	3.80 ± 0.09	3.93 ± 0.08	3.77 ± 0.08	0.149	0.180	0.106	0.100	NA
29 d*	3.83 ± 0.10	3.63 ± 0.06	3.76 ± 0.07	3.70 ± 0.08	3.81 ± 0.12	3.69 ± 0.06	0.528	0.792	0.241	0.662	NA
Ammonia [µg/dl]											
0.5-6.0 h	183.56 ± 11.28	157.38 ± 8.29	161.04 ± 9.68	179.33 ± 10.19	174.17 ± 16.42	168.26 ± 6.92	0.534	0.708	0.657	0.263	**0.050**
1 d	**168.29 ± 7.14^{a}**	**139.31 ± 5.85^{b}**	154.47 ± 7.16	153.20 ± 6.57	153.16 ± 8.93	154.11 ± 5.76	0.073	0.270	0.471	0.131	NA
4 d	146.15 ± 8.74	111.58 ± 6.24	120.51 ± 7.37	136.69 ± 8.37	119.39 ± 8.61	133.19 ± 7.19	0.338	0.574	0.800	0.992	NA
20 d	125.50 ± 10.83	106.43 ± 6.36	121.64 ± 9.87	110.04 ± 7.76	116.61 ± 10.03	115.22 ± 7.86	0.331	0.444	0.469	0.419	NA
29 d*	85.83 ± 5.04	80.75 ± 3.88	**75.35 ± 4.47^{A}**	**90.44 ± 4.14^{B}**	86.89 ± 5.71	81.52 ± 3.76	0.703	0.969	0.557	0.451	NA
Chloride [mmol/l]											
0.5-6.0 h	88.27 ± 0.89	90.23 ± 0.72	89.50 ± 0.85	89.04 ± 0.79	**88.57 ± 0.86§**	**89.61 ± 0.75$^{\$}$**	0.271	0.079	0.118	0.070	0.144
1 d	89.54 ± 1.17	87.38 ± 0.75	88.64 ± 0.99	88.29 ± 0.99	88.74 ± 1.31	88.32 ± 0.83	0.174	0.268	0.144	0.079	NA
4 d	90.02 ± 0.91	91.85 ± 0.95	91.30 ± 0.93	90.60 ± 0.94	**89.81 ± 1.15§**	**91.50 ± 0.80$^{\$}$**	0.145	0.080	**0.015**	**0.013**	NA

20 d	87.71 ± 1.43	90.53 ± 1.14	89.36 ± 1.52	88.98 ± 1.08	89.71 ± 1.58	88.92 ± 1.13	0.446	0.795	0.562	0.373	NA
29 d*	92.67 ± 1.60	94.62 ± 1.21	93.40 ± 1.44	93.98 ± 1.37	93.41 ± 2.02	93.82 ± 1.12	0.913	0.052	0.066	0.050	NA
Creatinine [mg/dl]											
0.5-6.0 h	**1.63 ± 0.08[a]**	**1.63 ± 0.06[b]**	**1.57 ± 0.08[A]**	**1.69 ± 0.07[B]**	1.65 ± 0.10	1.62 ± 0.06	**0.003**	0.717	0.184	0.340	0.519
1 d	**0.92 ± 0.03[a]**	**0.95 ± 0.03[b]**	**0.87 ± 0.03[A]**	**1.00 ± 0.03[B]**	0.97 ± 0.04	0.92 ± 0.03	**<0.001**	0.297	0.648	0.413	NA
4 d	**0.76 ± 0.03[a]**	**0.76 ± 0.02[b]**	**0.71 ± 0.03[A]**	**0.81 ± 0.02[B]**	0.72 ± 0.03	0.78 ± 0.02	**<0.001**	0.906	0.225	0.473	NA
20 d	0.85 ± 0.03	1.03 ± 0.05	0.86 ± 0.03	1.02 ± 0.05	0.97 ± 0.07	0.93 ± 0.03	0.518	0.568	0.118	0.823	NA
29 d*	0.98 ± 0.04	1.11 ± 0.04	**0.88 ± 0.04[A]**	**1.20 ± 0.03[B]**	1.05 ± 0.06	1.05 ± 0.04	0.602	0.185	0.142	0.235	NA
Fructose [mmol/l]											
0.5-6.0 h	2.03 ± 0.10	1.96 ± 0.15	2.06 ± 0.13	1.93 ± 0.12	1.80 ± 0.16	2.08 ± 0.11	0.902	0.115	0.670	0.448	**<0.001**
Haptoglobin [µg/ml]											
0.5-6.0 h	51.68 ± 7.94	35.07 ± 5.78	33.46 ± 5.27	53.29 ± 8.21	34.11 ± 7.69	47.50 ± 6.27	0.332	0.584	0.774	0.596	**<0.001**
1 d	**120.73 ± 10.78[a]**	**107.38 ± 6.47[b]**	102.29 ± 5.62	125.72 ± 11.05	124.34 ± 12.53	109.09 ± 7.13	0.056	**0.021**	**0.024**	**0.013**	NA
4 d	159.81 ± 26.23	159.50 ± 21.73	170.79 ± 22.29	148.74 ± 25.51	171.03 ± 32.03	154.14 ± 19.88	0.707	0.250	0.268	0.992	NA
20 d	337.84 ± 95.75	145.62 ± 23.02	333.67 ± 86.17	145.34 ± 41.44	254.26 ± 96.07	228.01 ± 54.22	0.674	0.098	**0.048**	0.096	NA
29 d*	474.22 ± 91.40	407.89 ± 21.58	461.31 ± 82.87	417.29 ± 34.52	509.33 ± 88.36	407.58 ± 49.94	0.700	0.792	0.395	0.864	NA
Sodium [mmol/l]											
0.5-6.0 h	**126.39 ± 0.97[a]**	**123.61 ± 0.64[b]**	125.19 ± 0.83	124.45 ± 0.79	**122.85 ± 0.87[§]**	**125.75 ± 0.71[$]**	0.897	**0.012**	0.340	0.095	0.330
1 d	**130.54 ± 0.92[a]**	**125.00 ± 0.59[b]**	**128.24 ± 0.94[A]**	**126.84 ± 0.75[B]**	125.97 ± 1.07	128.32 ± 0.71	0.424	0.704	**0.013**	0.214	NA
4 d	**129.86 ± 0.81[a]**	**126.02 ± 0.75[b]**	128.43 ± 0.88	127.33 ± 0.76	126.77 ± 1.07	128.41 ± 0.68	0.924	0.143	0.672	0.409	NA
20 d	120.24 ± 1.46	117.50 ± 0.83	119.47 ± 1.08	117.623 ± 1.11	117.70 ± 1.23	119.00 ± 0.99	**0.028**	**0.049**	**0.031**	**0.015**	NA
29 d*	121.34 ± 1.46	116.48 ± 0.84	119.13 ± 1.26	117.92 ± 1.07	**117.05 ± 1.71[§]**	**119.09 ± 0.93[$]**	0.531	**0.048**	**0.004**	**0.043**	NA
Urea [mg/dl]											
0.5-6.0 h	**11.90 ± 0.34[a]**	**11.37 ± 0.34[b]**	**11.58 ± 0.23[A]**	**11.69 ± 0.43[B]**	11.71 ± 0.42	11.60 ± 0.30	**0.002**	0.526	0.469	0.676	0.234
1 d	19.54 ± 0.91	17.36 ± 0.77	18.79 ± 0.83	18.13 ± 0.88	19.75 ± 1.16	17.84 ± 0.69	0.479	0.778	0.294	0.469	NA
4 d	8.36 ± 0.67	8.67 ± 1.03	9.26 ± 1.02	7.78 ± 0.70	**11.65 ± 1.29[§]**	**7.00 ± 0.59[$]**	0.424	0.847	0.669	0.458	NA
20 d	**5.91 ± 0.38[a]**	**8.23 ± 0.68[b]**	7.44 ± 0.51	6.80 ± 0.65	7.14 ± 0.55	7.10 ± 0.55	**0.041**	0.156	0.056	0.114	NA
29 d*	5.77 ± 0.44	6.74 ± 0.51	5.99 ± 0.49	6.56 ± 0.48	6.93 ± 0.59	6.00 ± 0.42	0.804	0.221	0.763	0.686	NA
Uric acid [mg/dl]											
0.5-6.0 h	**0.43 ± 0.01[a]**	**0.54 ± 0.02[b]**	0.48 ± 0.02	0.49 ± 0.02	0.52 ± 0.03	0.47 ± 0.02	0.366	0.540	0.369	0.747	0.523
1 d	0.48 ± 0.02	0.48 ± 0.01	0.47 ± 0.02	0.49 ± 0.01	0.51 ± 0.03	0.47 ± 0.01	0.968	0.685	0.768	0.592	NA
4 d	0.55 ± 0.03	0.63 ± 0.05	0.65 ± 0.05	0.53 ± 0.02	0.74 ± 0.08	0.52 ± 0.02	0.608	0.802	0.661	0.541	NA
20 d	0.30 ± 0.01	0.34 ± 0.01	0.32 ± 0.01	0.33 ± 0.01	0.33 ± 0.01	0.32 ± 0.01	0.942	0.479	0.249	0.275	NA

29 d*	0.30 ± 0.01	0.34 ± 0.01	0.32 ± 0.01	0.32 ± 0.01	0.33 ± 0.01	0.32 ± 0.01	0.807	0.692	0.452	0.622	NA

* post-weaning; NA: not available;

Samples were taken at 0.5-6.0 h, day 1, day 4, day 20, and day 29. The Data were collected from selected German Landrace-piglets (n = 96) classified to birth weight classes from induced and spontaneous farrowings in farrowing crate and farrowing pens. Data are presented as mean ± SEM. Significant effects are highlighted in bold (P < 0.05).

Different superscripts indicate statistical significance per individual time point (P < 0.05) within parturition induction (^{a},^{b}), within farrowing environment (^{A},^{B}), and within birth weight class (§,$^{\$}$).
