# Supplementary material for: Impact of parturition induction, farrowing environment and birth weight class on endocrine and metabolic plasma parameters related to piglet vitality
Source: BMC Vet Res. 2025 Jun 7;21:406. doi: 10.1186/s12917-025-04845-2 (PMC12144723; doi:10.1186/s12917-025-04845-2)
Supplement: Supplementary file 4 — Supplementary Material 4 [file 12917_2025_4845_MOESM4_ESM.pdf]

Impact of partus induction, housing system and birth weight class on endocrine and metabolic plasma parameters related to piglet vitality

H Lickfett[1,2], M Oster[1], A Vernunft[1], H Reyer[1], E Muráni[1], S Görs[1], CC Metges[1], H Bostedt[2], K Wimmers[1,3,*]

[1]Research Institute for Farm Animal Biology (FBN), 18196 Dummerstorf, Germany;

[2]Veterinary Clinic for Reproductive Medicine and Neonatology, Justus-Liebig-University Gießen, 35392 Gießen, Germany;

[3]Chair of Animal Breeding and Genetics, Faculty of Agricultural and Environmental Sciences, University Rostock, 18059 Rostock, Germany;

*Correspondence: Email: wimmers@fbn-dummerstorf.de; Tel.: +49-38208-68-600;

Supplemental Table S2: Overview of plasma parameters analysed at selected time points during the study period.

Parameter	0.5-6.0 h	1 d	4 d	20 d	29 d*
Weight	×	×	×	×	×
Albumin	×	×	×	×	×
Ammonia	×	×	×	×	×
Chloride	×	×	×	×	×
Cortisol	×	×	×	×	×
Creatinine	×	×	×	×	×
Fructose	×				
Glucose	×	×	×		
Haptoglobin	×	×	×	×	×
Inositol	×	×			
Insulin	×	×	×		
Lactate	×	×	×		
NEFA	×	×	×	×	×
Sodium	×	×	×	×	×
Total protein	×	×	×	×	×
Triglycerides	×	×	×	×	×
Triiodothyronine	×		×		
Urea	×	×	×	×	×
Uric acid	×	×	×	×	×

* post-weaning; NEFA: non-esterified fatty acids;

Due to our commitment to 3R principle, the available plasma volume was limited and the analysed parameters had to be prioritized.
