# Supplementary material for: Impact of parturition induction, farrowing environment and birth weight class on endocrine and metabolic plasma parameters related to piglet vitality
Source: BMC Vet Res. 2025 Jun 7;21:406. doi: 10.1186/s12917-025-04845-2 (PMC12144723; doi:10.1186/s12917-025-04845-2)
Supplement: Supplementary file 5 — Supplementary Material 5 [file 12917_2025_4845_MOESM5_ESM.pdf]

Impact of partus induction, housing system and birth weight class on endocrine and metabolic plasma parameters related to piglet vitality

H Lickfett[1,2], M Oster[1], A Vernunft[1], H Reyer[1], E Muráni[1], S Görs[1], CC Metges[1], H Bostedt[2], K Wimmers[1,3,*]

[1]Research Institute for Farm Animal Biology (FBN), 18196 Dummerstorf, Germany;

[2]Veterinary Clinic for Reproductive Medicine and Neonatology, Justus-Liebig-University Gießen, 35392 Gießen, Germany;

[3]Chair of Animal Breeding and Genetics, Faculty of Agricultural and Environmental Sciences, University Rostock, 18059 Rostock, Germany;

*Correspondence: Email: wimmers@fbn-dummerstorf.de; Tel.: +49-38208-68-600;

Supplemental Table S3: Plasma metabolites covering the energy metabolism of neonates and suckling piglets.

Time point	Parturition induction		Farrowing environment		Birth weight class		Interactions				*P*-value time between first suckling and blood sampling
	Induced	Spontaneous	Crate	Pen	800-1100g	>1100g	Birth induction × farrowing environment	Birth induction × birth weight class	Farrowing environment × birth weight class	Birth induction × farrowing environment × birth weight class	
Cortisol [ng/ml]											
0.5-6.0 h	91.45 ± 8.74	97.14 ± 10.71	95.91 ± 10.53	92.70 ± 8.97	112.77 ± 12.90	85.81 ± 7.98	0.136	0.712	0.398	0.229	0.986
1 d	123.19 ± 12.71	79.10 ± 6.27	111.39 ± 10.69	91.56 ± 10.29	**119.63 ± 16.35[§]**	**92.39 ± 7.51[$]**	0.231	**0.027**	0.545	0.490	NA
4 d	71.28 ± 7.13	39.59 ± 5.11	74.75 ± 7.68	36.04 ± 3.47	**69.77 ± 10.27[§]**	**48.64 ± 4.65[$]**	0.184	0.194	0.086	0.275	NA
20 d	35.04 ± 4.88	24.90 ± 3.66	37.58 ± 4.70	22.52 ± 3.70	28.62 ± 4.15	30.33 ± 4.03	0.828	0.177	0.126	0.384	NA
29 d*	32.71 ± 4.46	43.34 ± 3.87	44.52 ± 4.82	32.53 ± 3.41	41.04 ± 4.79	37.14 ± 3.74	0.205	0.843	0.425	0.515	NA
Glucose [mmol/l]											
0.5-6.0 h	5.91 ± 0.35	5.30 ± 0.29	6.14 ± 0.33	5.06 ± 0.30	4.97 ± 0.36	5.89 ± 0.29	0.924	0.649	0.515	0.822	0.189
1 d	**5.88 ± 0.27[a]**	**5.84 ± 0.14[b]**	**6.21 ± 0.24[A]**	**5.53 ± 0.18[B]**	**5.36 ± 0.27[§]**	**6.11 ± 0.18[$]**	**0.040**	**0.020**	0.381	0.112	NA
4 d	**7.02 ± 0.25[a]**	**6.40 ± 0.15[b]**	**7.04 ± 0.23[A]**	**6.38 ± 0.18[B]**	**6.46 ± 0.28[§]**	**6.82 ± 0.17[$]**	0.439	0.056	0.060	0.279	NA
Lactate [mmol/l]											
0.5-6.0 h	4.28 ± 0.22	4.28 ± 0.36	4.33 ± 0.37	4.22 ± 0.20	4.22 ± 0.27	4.31 ± 0.28	0.427	0.649	0.964	0.757	0.293
1 d	5.58 ± 0.30	4.83 ± 0.30	**5.68 ± 0.33[A]**	**4.76 ± 0.25[B]**	5.15 ± 0.33	5.24 ± 0.27	0.112	0.678	0.779	0.225	NA
4 d	**6.55 ± 0.38[a]**	**4.52 ± 0.25[b]**	5.51 ± 0.37	5.53 ± 0.34	5.15 ± 0.41	5.70 ± 0.31	0.770	0.107	0.116	0.228	NA
Inositol [mmol/l]											
0.5-6.0 h	4.41 ± 0.28	4.67 ± 0.36	**5.03 ± 0.33[A]**	**4.02 ± 0.30[B]**	5.67 ± 0.33	4.01 ± 0.27	0.945	0.684	0.256	0.741	**0.001**
1 d	3.03 ± 0.20	2.88 ± 0.18	**3.00 ± 0.19[A]**	**2.91 ± 0.19[B]**	3.51 ± 0.23	2.67 ± 0.15	0.132	0.593	**0.021**	0.221	NA
Insulin [mU/l]											
0.5-6.0 h	31.09 ± 4.67	28.98 ± 5.40	29.20 ± 5.07	31.02 ± 4.99	18.08 ± 4.22	34.74 ± 4.54	0.214	0.106	0.090	0.071	**0.034**

1 d	9.85 ± 1.95	14.55 ± 2.43	16.15 ± 2.75	8.80 ± 1.51	13.75 ± 3.58	11.86 ± 1.71	0.225	0.173	0.734	0.515	NA
4 d	11.21 ± 1.59	9.24 ± 1.26	12.49 ± 1.56	7.58 ± 1.07	10.13 ± 1.69	10.13 ± 1.24	0.638	0.644	0.828	0.603	NA
NEFA [µmol/l]											
0.5-6.0 h	107.612 ± 9.41	100.82 ± 11.52	109.77 ± 11.15	98.52 ± 9.89	87.57 ± 13.20	111.92 ± 8.91	0.418	0.458	0.967	0.414	**<0.001**
1 d	125.51 ± 5.63	146.33 ± 7.18	139.27 ± 5.54	132.43 ± 7.46	147.33 ± 10.24	130.15 ± 4.67	0.773	0.231	0.806	0.859	NA
4 d	**223.13 ± 15.60[a]**	**264.54 ± 16.16[b]**	237.00 ± 18.87	250.96 ± 13.01	**288.94 ± 25.51[§]**	**222.31 ± 10.66[$]**	0.341	0.137	0.546	0.338	NA
20 d	275.91 ± 26.95	284.66 ± 16.45	289.26 ± 26.98	272.08 ± 15.78	285.25 ± 22.63	278.33 ± 19.99	0.283	0.732	0.857	0.713	NA
29 d*	335.81 ± 53.66	414.05 ± 65.20	339.17 ± 59.95	412.61 ± 60.89	588.83 ± 97.52	284.94 ± 39.40	0.906	0.755	0.762	0.693	NA
Total protein [g/dl]											
0.5-6.0 h	2.69 ± 0.10	2.58 ± 0.08	2.48 ± 0.07	2.79 ± 0.10	2.53 ± 0.12	2.68 ± 0.08	0.307	0.421	**0.045**	0.099	**<0.001**
1 d	4.65 ± 0.13	5.37 ± 0.11	4.98 ± 0.13	5.04 ± 0.14	4.81 ± 0.18	5.10 ± 0.10	0.076	0.481	0.757	0.974	NA
4 d	**4.44 ± 0.08[a]**	**5.09 ± 0.08[b]**	4.74 ± 0.09	4.79 ± 0.09	4.62 ± 0.11	4.83 ± 0.08	0.343	0.839	0.632	0.750	NA
20 d	**4.53 ± 0.15[a]**	**4.63 ± 0.070[b]**	4.58 ± 0.08	4.58 ± 0.13	4.53 ± 0.08	4.60 ± 0.11	**0.049**	0.064	0.037	**0.014**	NA
29 d*	4.45 ± 0.11	4.46 ± 0.06	4.53 ± 0.07	4.38 ± 0.01	4.47 ± 0.14	4.45 ± 0.06	0.625	0.605	0.370	0.974	NA
Triglyceride [mg/dl]											
0.5-6.0 h	35.07 ± 4.32	23.23 ± 3.47	**29.27 ± 3.59[A]**	**28.65 ± 4.35[B]**	24.35 ± 5.42	31.11 ± 3.25	0.916	0.676	0.109	0.749	**<0.001**
1 d	51.27 ± 5.51	66.63 ± 5.87	64.17 ± 6.02	53.94 ± 5.50	**56.00 ± 7.77[§]**	**60.35 ± 4.79[$]**	0.712	0.094	0.213	0.411	NA
4 d	168.53 ± 10.22	148.77 ± 9.81	158.11 ± 10.37	159.00 ± 9.87	178.36 ± 15.17	148.95 ± 7.39	0.598	0.532	0.471	0.201	NA
20 d	63.27 ± 4.51	58.81 ± 4.01	61.21 ± 4.29	60.75 ± 4.25	61.68 ± 6.03	60.65 ± 3.44	0.237	0.757	0.242	0.822	NA
29 d*	39.15 ± 4.26	40.36 ± 3.61	43.55 ± 4.41	36.37 ± 3.36	48.63 ± 5.58	35.89 ± 3.01	0.700	0.723	0.904	0.333	NA
Triiodothyronine [ng/ml]											
0.5-6.0 h	3.92 ± 0.43	2.73 ± 0.22	3.10 ± 0.26	3.56 ± 0.43	2.93 ± 0.26	3.53 ± 0.35	0.547	0.709	0.798	0.786	**<0.001**
4 d	6.09 ± 0.55	4.66 ± 0.28	5.67 ± 0.41	5.03 ± 0.46	5.04 ± 0.47	5.50 ± 0.40	0.213	0.643	0.285	0.603	NA

* post-weaning; NEFA: non-esterified fatty acids; NA: not available;
Samples were taken at 0.5-6.0 h, day 1, day 4, day 20, and day 29. The data were collected from selected German Landrace-piglets (n = 96) classified to birth weight classes from induced and spontaneous farrowings in farrowing crates or farrowing pens. Data are presented as mean ± SEM. Significant effects are highlighted in bold (*P* < 0.05).
Different superscripts indicate statistical significance per individual time point (*P* < 0.05) within parturition induction ([a],[b]), within farrowing environment ([A],[B]), and within birth weight class ([§],[$]).
